# Challenges in the evaluation of HER2 and HER2-low in breast cancer in Brazil and recommendations of a multidisciplinary working group

**DOI:** 10.1590/1806-9282.20240313

**Published:** 2024-09-30

**Authors:** Helenice Gobbi, Filomena Marino Carvalho, Marina De Brot, Angela Flavia Logullo, Carlos Augusto Moreira Silva, Fernando Augusto Soares, Luciana Landeiro, Rosemar Rahal, Carlos Henrique Barrios

**Affiliations:** 1 Universidade Federal do Triângulo Mineiro, Discipline of Special Pathology – Uberaba (MG), Brazil.; 2 Universidade de São Paulo, Department of Pathology – São Paulo (SP), Brazil.; 3 A.C.Camargo Cancer Center, Department of Pathological Anatomy – São Paulo (SP), Brazil.; 4 Universidade Federal de São Paulo, Department of Pathology – São Paulo (SP), Brazil.; 5 Institute of Molecular and Surgical Pathology - Ophir Loyola Hospital – Belém (PA), Brazil.; 6 Universidade de São Paulo, Institute of Pathological Anatomy, School of Dentistry, D´Or São Luiz Network – São Paulo (SP), Brazil.; 7 Oncoclínicas Group – Salvador (BA), Brazil.; 8 Universidade Federal de Goiás, Department of Gynecology – Goiânia (GO), Brazil.; 9 Latin American Cooperative Oncology Group and Oncoclínicas Group – Porto Alegre (RS), Brazil.

## INTRODUCTION

Currently, breast cancer (BC) is the most common cancer among women worldwide, accounting for more than 2.3 million cases annually^1^, and the leading cause of cancer death in women^2^.

Between 1989 and 2017, a substantial 40% reduction in mortality from BC was observed in developed countries; however, this is not yet the case in developing countries such as Brazil, where the mortality rate of BC is approximately 60%^3^.

BC is classified according to its gene expression profile into five different tumor subtypes, referred to as intrinsic molecular subtypes: luminal A, luminal B, human epidermal growth factor receptor 2 (HER2)-positive, basal-like, and a group referred to as normal-breast cancer-like^4^. The specific subtype of BC has an impact on the prognosis and treatment of the disease^5^.

Due to the high cost of differentiating among those five subtypes using gene expression profiles, in clinical practice, immunohistochemistry (IHC) is used to classify BC according to the protein expression levels of estrogen and progesterone receptors and HER2 [the latter of which is also assessed with in situ hybridization (ISH)] into hormone receptor-positive, HER2-positive, and triple-negative BC. Among the BC subtypes, “pure” HER2-positive tumors (defined by IHC classification as a HER2 score of 3+ or 2+ with ERBB2 amplification by ISH)^6,7^ account for approximately 15–20% of all cases^8^ and exhibit more aggressive behavior than HER2-negative tumors^5^.

With the introduction of therapies based on anti-HER2 monoclonal antibodies, such as trastuzumab and pertuzumab, approximately two decades ago, the life expectancy of HER2-positive patients has significantly improved^8,9,10,11,12^. However, most patients (80–85%) with BC are HER2-negative (defined by an IHC staining score of 0 and 1+ or an IHC staining score of 2+ with negative ISH findings)^6^, for whom these anti-HER2 therapies are not efficacious^5^.

Recently, with the emergence of monoclonal antibody-drug conjugate (ADC) treatments, such as trastuzumab deruxtecan (T-DXd), the evaluation of this group of HER2-negative patients is in a state of flux^13^. T-DXd consists of a compound in which a molecule of trastuzumab (an anti-HER2 antibody) binds to a molecule (linker) and incorporates a certain number of molecules from a potent cytotoxic agent (payload)^14,15,16,17^. The main study that showed the benefit of T-DXd in tumors with low HER2 expression included patients with IHC scores of 1+ and 2+/ISH-negative; these patients were termed the HER-2-low subgroup. Patients with an expression lower than a score of 1+, i.e., weak partial expression in membranes in up to 10% of the cells, and those with a score of 0 together and completely HER2-negative patients, were excluded from that study^5^.

Thus, accurately identifying HER2 expression in BC patients in categories 0, 1+, 2+, and 3+ is essential for recommending the appropriate treatment for the patient. For this purpose, a multidisciplinary study is essential to ensure the quality of tumor samples (needle biopsy or surgical specimens), adequate logistics until sample arrival at the pathology laboratory, and accurate evaluation of histopathology, IHC, and molecular characteristics^18^.

In Brazil, this entire complex process, from the collection of tissue samples to the final analysis performed by pathologists, faces important challenges related to organizational, economic, and communication gaps that negatively impact the accurate determination of the HER2 tumor status^18^.

In this article, a multidisciplinary group of breast cancer specialists identified and discussed relevant aspects of the entire sample journey (SJ) patients with BC in Brazil today, with the objective of reviewing existing recommendations based on available scientific evidence and improving preanalytical, analytical, and postanalytical processes in the diagnosis of BC and the determination of HER2 status.

## METHODOLOGY

### Working group

The working group was composed of individuals from three different specialties: six pathologists, two clinical oncologists, and one breast cancer surgeon. The members of the group work in clinics and hospitals in both the public and private sectors located in different regions of Brazil and have extensive experience in BC.

### Project phases

This project was divided into three phases, as described below:

Phase 1: Mapping of obstacles and challenges in the preanalytical, analytical, and postanalytical processes.

Phase 2: Discussion of the main obstacles encountered.

Phase 3: Multidisciplinary elaboration of recommendations.

The process mapping phase was performed using a specific questionnaire developed by the project leaders (a clinical oncologist and a pathologist) and answered by the specialists of the working group as well as six (nonacademic) community pathologists. In all, 12 pathologists from different states of Brazil (Rondônia, Pará, Goiás, Federal District, Rio Grande do Sul, Minas Gerais, and São Paulo), 2 clinical oncologists, and 1 breast cancer surgeon answered the questionnaires with the objective of identifying the realities encountered by women from different regions in Brazil during the sample journey.

In the second and third phases, to discuss the obstacles/challenges encountered and make appropriate, we analyzed and discussed the responses to the questionnaire with all members of the group and proposed improvements in all phases of the sample journey via the formulation of recommendations for clinical practice.

## RESULTS

The responses to the questionnaires were evaluated, and the barriers and challenges were identified in the three phases of the sample journey (preanalytical, analytical, and postanalytical), as detailed in Table 1.

**Table 1:** Obstacles and challenges encountered during the preanalytical, analytical, and postanalytical phases of the sample journey and recommendations made by the working group.

Phase of the sample journey	Obstacles/challenges	Recommendation of the working group
Preanalytical phase	Lack of information in the examination request.	Foster education for professionals involved; follow a medical request model with essential information^a^.
	Inadequate cold ischemia time/quality of fixation, decalcification process.	Conduct continuing education of the treatment team; management consulting and acquisition of inputs^a^.
	Failure to follow the recommendations of sample care by surgeons, radiologists, and pathology services.	Coordinators, managers, and pathologists of the services involved should provide adequate tools for continuing education and periodic training^a^.
	Logistics involved until the arrival of the sample at the pathology laboratory.	Appropriate transportation methods, timing and packaging to maintain the quality of the fixation within the recommended parameters, not exceeding a maximum period of 72 h^a^.
	Nonparticipation in external accreditation and quality programs by the laboratories.	Encourage participation in continuing education programs and external laboratory accreditation and quality control programs in immunohistochemistry^b^.
Analytical phase	Lack of procedure standardization and internal validation.	Perform standardization and internal validation of all tests and receipt of all new kits.
	Use of different platforms, types of antibodies, concentrations, and detection systems.	Preferably use automated platforms. When manual platforms are used, quality reagents with standardization and validation should be employed.
	No controls with different levels of HER2 expression (0, 1+, 2+, and 3+).	Include controls for different HER2 expression scores in all reactions, preferably on the same slide.
	Lack of participation in external accreditation and quality control programs.	Encourage participation in external laboratory accreditation and quality control programs in immunohistochemistry.
	Lack of training of the technical staff.	Encourage participation in training and continuing education programs periodically.
	Time and storage conditions of paraffin blocks.	Store blocks under appropriate conditions for the recommended time^c^.
Postanalytical phase	Some laboratories did not use the HER2 classification established by the ASCO/CAP in the reports, using only binary classification (positive versus negative).	Follow the 2023 ASCO/CAP guidelines and include categories 0, 1+, 2+, and 3+ in the immunohistochemical reports. We suggest the inclusion of a note in cases of low and ultra-low HER2 expression (HER2 >0 and <1+, 1+) and in cases with scores of 2+ and unamplified ISH^d^.
	Lack of information in the report about the type of antibody (clone) and platform used.	Include the information in the report.
	Lack of description in the reports of HER2 expression heterogeneity in the tumor.	Include the information in the report.
	Difficulties in authorizing procedures that involve biopsies and reanalysis by pathology to evaluate HER2 during the course of the disease.	Inform the paying sources about the possible changes in the expression of markers and the need for retesting during the course of the disease.
	Lack of discussion and review by another pathologist for cases of borderline low and ultra-low HER2 expression (1+ and >0<1+).	Creation of double-checking processes/flows by pathologists in challenging and low-expression cases^c^.
	Little multidisciplinary interaction for case discussion.	Stimulate the discussion of cases among specialists and hold multidisciplinary meetings with the participation of the pathologist. Make managers aware of the importance of the pathologist and of his or her participation in these discussions.
	Lack of updating and training for the correct analysis and interpretation of the immunohistochemical test.	Encourage participation in training and continuing education courses for test interpretation.

^a^Recommendations that already exist in the guidelines of the Quality Control Accreditation Program of the Brazilian Society of Pathology (PACQ/SBP), College of American Pathologists (CAP), and United Kingdom National External Quality Assessment Scheme (UK-NEQAS)^23,25,28^.

^b^Existing recommendations made by SBP/PACQ^24,25^.

^c^Existing recommendations made by ANVISA and the Brazilian Society of Pathology^29^.

^d^Existing recommendations made by the American Society of Clinical Oncology (ASCO) and CAP^28^.

## DISCUSSION AND RECOMMENDATIONS

### Preanalytical phase

The preanalytical phase is of particular importance as it influences the subsequent steps. In this phase of sample collection and initial processing, different professionals, such as physicians of various specialties, nurses, nursing technicians, administrative employees, and transport personnel, are involved^19^.

Evidence indicates that 31–75% of errors related to pathology laboratory tests occur in this phase^20^. The most common errors are in basic processes such as correct patient registration and identification, sample collection and preparation, and transport and storage^19,21^.

In Brazil, a relevant and frequent issue is that the samples are generally sent to pathology laboratories outside the collection site, either because the hospital does not have its own pathology service or due to physician preference^22^. The logistics involved in this process, both due to the internal organization of the surgical center and the distance between the hospital and the pathology laboratory, can be challenging. The time required for a sample to reach the laboratory for sectioning and analysis can be very long, ranging from 2 h to weeks.

In addition, factors such as the time between tumor sample removal and fixation (time to cold ischemia), the type of fixative used, and the duration of fixation impact the subsequent phases. The sample must be sectioned immediately after removal and immersed in buffered formalin for fixation for a maximum period of 48 h^18,22^.

Delayed sectioning and prolonged fixation may interfere with the diagnosis^18,22^.

Some Brazilian laboratories do not follow the recommendations of the American Society of Clinical Oncology/College of American Pathologists (ASCO/CAP)^6^, the United Kingdom National External Quality Assessment Scheme (UK-NEQAS)^23^, or the Brazilian Pathology Society Quality Control Accreditation Program (Programa de Acreditação e Controle da Qualidade da Sociedade Brasileira de Patologia, SBP/PACQ)^24,25^ for the preanalytical stage (recording the time the material was obtained and immersed in the fixative, sectioning, fixation, type, and duration of fixation), and most do not participate in laboratory accreditation or external quality control programs for preanalytical procedures.

The recommendations made by this working group for the main obstacles/challenges identified in the preanalytical phase are described in Table 1.

### Analytical phase

The analytical phase involves the receipt of the material in the pathology laboratory, macroscopic examination, sectioning, tumor sample selection, technical processing, embedding in paraffin, microtomy, routine staining, and IHC^26^.

Insufficient or excessive fixation may impair the detection of protein immunoexpression, and eventually, IHC may not be sufficiently sensitive to accurately detect low and ultra-low levels of HER2 expression^27^.

Several intralaboratory factors can affect the result of HER2 status evaluation, such as lack of procedure standardization and internal validation, use of nonautomated immunohistochemical methods, and lack of operator training and/or adequate equipment programming for automated methods. In addition, the use of different types of antibodies (monoclonal or polyclonal) and different concentrations therein can lead to different results^18,28^.

Another point emphasized is the tissues subjected to decalcification in an acid medium, as this may result in the variable loss of antigenic sites, leading to decreased protein detection^6^.

Another factor to be considered is the duration of paraffin block storage. Fortunately, today, patients survive longer, and new drugs have been developed; this has generated a demand for research on biomarkers involving tumors that had been previously diagnosed and stored in paraffin blocks. One example involves examining the HER2 expression pattern; under certain circumstances, the diagnosis needs to be made on material collected and stored years ago. It has been shown that for paraffin blocks of samples that have been properly fixed, DNA analysis can be performed up to 5 years later, RNA analysis up to 1 year later, and protein evaluation via IHC up to 25 years later^29^. Therefore, even if legislation allows shorter storage periods, longer storage periods should be considered.

In addition to the standardization of procedures and the quality of the reagents, the analytical phase depends on the preanalytical stage, training for the analysis and correct interpretation of the tumor sample (attentive and careful analysis regarding the standards of membrane staining, percentage of labeled invasive neoplastic cells, and identification artifacts), and adequate communication between the professionals involved in handling the samples (radiologists, breast cancer surgeons, oncology surgeons, pathologists, and clinical oncologists). The lack of integration among the team members involved in the entire sample journey can result in the loss of important information, which can have a significant impact on the interpretation of the results^18,22^.

Finally, a significant factor that has a negative impact on the entire process is the lack of participation in external accreditation or quality control programs. Improvements in all these processes may improve the identification of HER-2-low patients and, consequently, expand therapeutic options^27^.

The recommendations made by the working group for addressing the main obstacles/challenges identified in the analytical phase are described in Table 1.

### Postanalytical phase

The postanalytical phase involves IHC staining analysis and ends with the immunohistochemical study report^19^. It is important to evaluate the quality of the sample; inadequacies should be included in the final report^18^.

Some laboratories do not use the HER2 classification recommended by international consensus (e.g., reporting scores of 0, 1+, 2+, and 3+); rather, according to the questionnaire responses and the discussion among the experts, only a binary classification (positive versus negative) is used. Accurate evaluation of HER2 expression in breast cancer is important for properly determining the therapeutic regimen that should be prescribed to the patient by the oncologist. According to the recommendations of the ASCO/CAP 2018 and 2023 committees, distinguishing and referencing the specific score is mandatory^6,28^.

A thorough analysis of the intensity and patterns of membrane staining, the percentage of labeled neoplastic cells, and the recognition of artifacts is essential. For these analyses, high-magnification microscopes (400×) are particularly useful. Borderline cases (those between a score of 0 and 1+) should be discussed and reviewed by other colleagues, and dubious cases (score 2+) or those with less common staining patterns should be further subjected to in situ hybridization for evaluating the HER2 status, as recommended by the 2023 update of the ASCO/CAP consensus^28^.

Attention should also be paid to the identification of overstaining (moderate/strong membrane staining in non-neoplastic cells), fulguration/crushing artifacts, edge artifacts, retraction artifacts with diaminobenzidine condensation around the spaces, simulated membrane labeling, and exclusion of carcinoma in situ foci^28^.

Other relevant points observed in the postanalytical phase were a lack of information in the report about the type of antibody (clone) used and the lack of description of the presence of tumor heterogeneity.

Currently, despite being defined as HER2-negative, breast cancer tumors with low HER2 expression have detectable amounts of HER2 protein in the cell membranes (IHC score of 1+ or 2+/nonamplified ISH)^27^. Recent evidence indicates that new anti-HER2 agents are effective against tumors that have low HER2-receptor expression, emphasizing the importance of identifying tumors with low (1+ or 2+/nonamplified ISH) or ultra-low (<1+ and >0) expression of the HER2 protein^30^.

Both low HER2 expression and HER2 expression heterogeneity in a given tumor affect the therapeutic response. The definition of HER2-positive status is based on intense, uniform, and circumferential expression in the entire cell membrane in more than 10% of the tumor cells. Heterogeneous expression patterns have been described with clustered, mosaic, or dispersed distributions. To detect intratumoral genetic heterogeneity, in situ hybridization testing is recommended^31^. Although intratumoral heterogeneity has generally been associated with a worse therapeutic response, the clinical significance of these different patterns (protein and/or nongenetic heterogeneity) has not yet been validated^32^.

The expression of HER2, particularly that of the HER-2-low phenotype, appears to vary over time and between primary tumor and metastatic lesions. The prognosis also varies according to the organ and even to the biopsy site. A recent study suggested that new biopsies are able to identify HER-2-low expression in 100% of patients after only five biopsies are performed^33,34^.

It was also highlighted in the Brazilian services evaluated by the working group that when there is a need for rebiopsy and/or reanalysis of sample blocks or biopsy of metastatic sites, it is often difficult to obtain authorization from the paying source (supplementary system).

Other problems observed were a lack of consensus or knowledge about the appropriate storage time for reviewing slides from archived blocks; a lack of discussion and review of cases by two pathologists for borderline or low HER2 expression tumors; and a lack of discussion of cases among pathologists, oncologists, and breast specialists. Importantly, there are differences in test results depending on the pathologist^35,36^. One study examined the determination of HER2 scores of 0, 1+, and 2+ versus 3+ and identified a discrepancy of 11%. When comparing HER2 scores of 0 versus 1+, 2+, and 3+, the discrepancy reached 41%^36^. Thus, the training of pathologists to evaluate the HER2 expression spectrum is important and would have therapeutic value. Viale et al. demonstrated improvement in diagnostic quality after pathologist training. The authors found that >30% of HER2 0 tumors changed to HER-2-low after training, while only <10% of HER-2-low were reassigned a score of 0^37^.

Recently, it was proposed that tumors with very low protein expression (score of zero with incomplete staining and weak staining in ≤ 10% of tumor cells) be referred to as “ultra-low” HER2^8^. However, the 2023 ASCO/CAP Consensus does not recommend the use of this terminology in pathological reports but justified its use by oncologists for therapeutic decision-making. It is important to note that the updated recommendations of the committee included the specification of the “absence of HER2 overexpression” in cases scored 0, 1+, and 2+/ISH without gene amplification in the classification nomenclature, in addition to discussing the addition of a note in the reports about the concept of HER-2-low and its prognostic significance^28^.

HER2 overexpression indicates that the disease is biologically dependent on receptor signaling. Thus, receptor blockade has a biological and therapeutic impact, as has been widely demonstrated in previous studies. In tumors with low HER2 expression, including those in the HER-2-low subgroup, this phenomenon does not occur; consequently, there is no benefit from blocking the HER2 pathway^10,11^. The rationale for the use of ADCs in low HER2-expression cancer is that the use of an antibody against a differentially expressed molecule in the neoplasm increases the concentration of the cytotoxic agent in the tumor area. For new ADCs, such as T-DXd, the linker between the antibody and the payload is cleavable and soluble, allowing the “bystander” effect in which the cytotoxic agent is released into the tumor microenvironment after killing the cell, thus affecting neighboring cells. This could explain the benefits of these new agents both in tumors that heterogeneously express HER2 and in those with low HER expression, in which the receptor is a mere conductor of the antibody with its toxic load^38^.

More precise and reproducible methods for the determination of HER2 expression are needed. The IHC method was developed to identify patients with protein overexpression who are more likely to respond to anti-HER2 therapies, not to differentiate high versus low and ultra-low expression. Further standardization of the IHC method and pathologist training is necessary for detecting very low levels of HER2 expression, as is the use of new methods that allow the more accurate quantification of different levels of protein expression.

New methodologies are being proposed and tested to qualify our ability to differentiate these patients and assist in therapeutic selection^39,40^.

The recommendations made by the working group for the main obstacles/challenges identified in the postanalytical phase are described in Table 1.

## CONCLUSION

Although the three phases involved in HER2 testing in breast cancer are important, the main challenges in the practical implementation of the recommendations of this consensus in Brazil are in the preanalytical phase that involves fixing, transporting to the laboratory, and sectioning of surgical specimens to avoid protein damage resulting from cold ischemia. Another challenge is the analysis of immunohistochemical reactions. Brazilian pathologists must now be trained in distinguishing the entire spectrum of HER2 expression, including cases with low and ultra-low HER2 expression, with therapeutic implications for patients. The adoption of the actions recommended by this working group will involve the education and training of everyone involved in the three stages of the process.
